# Identification of Peristomal Skin Alterations Using Convolutional Artificial Neural Networks

**DOI:** 10.1155/nrp/2927548

**Published:** 2026-06-14

**Authors:** Isabel María López-Medina, César Hueso-Montoro, Francisco Charte-Ojeda, Carmen Álvarez-Nieto, José Pablo Soriano-Torres, Francisco Pedro García-Fernández, Concepción Capilla-Díaz, Ana Carmen Montesinos-Gálvez, Noelia Moya-Muñoz, Claudia Cuevas-Sánchez, María Dolores Pérez-Godoy

**Affiliations:** ^1^ Department of Nursing, University of Jaén, Jaén, Spain, ujaen.es; ^2^ IT Department, University of Jaén, Jaén, Spain, ujaen.es; ^3^ Department of Nursing, University of Granada, Granada, Spain, ugr.es; ^4^ Clinical Management Unit for General Surgery, Gastroenterology and Liver Transplantation, Regional University Hospital, Málaga, Spain; ^5^ Neurotrauma ICU, Virgen de Las Nieves University Hospital, Granada, Spain, hvn.es; ^6^ PhD School, University of Jaén, Jaén, Spain, ujaen.es

**Keywords:** artificial intelligence, convolutional neural networks, peristomal skin, stoma nurse

## Abstract

**Introduction:**

Peristomal skin complications (PSCs) are very common among ostomy patients and significantly affect their quality of life and healthcare costs. Although convolutional neural networks (CNNs) offer possibilities for automated diagnosis, specific AI applications for the treatment of PSCs are underdeveloped.

**Aims:**

To develop and validate preliminary models based on CNNs for the binary classification of peristomal skin, enabling the distinction between healthy tissue and the presence of skin lesions, thereby laying the foundations for automated diagnostic systems.

**Design:**

Prospective study.

**Methods:**

The data and images were collected by 24 stoma nurses from 17 hospitals participating in the study. We addressed the classification of peristomal skin images using state‐of‐the‐art pretrained CNNs. The classification models were evaluated using the measures accuracy, F1‐score, and the area under the ROC curve. Finally, the Grad‐Cam explainability algorithm is applied to the best model.

**Results:**

With 1165 images collected, several models were tested. The data were split using standard 10‐fold cross‐validation. A dual experiment was conducted. First, a standard data split was employed, yielding an accuracy of 0.889, an F1‐score of 0.890, and an area under the ROC curve of 0.924 for the best model. Second, the data were split so that images from the same patient would not be distributed across the training and test subsets, thereby preventing data leakage. The best results for this experiment were 0.778, 0.868, and 0.653, respectively.

**Conclusions:**

By processing peristomal skin images with artificial intelligence, we developed robust, reliable, preliminary models for detecting peristomal skin alterations. The models allow the automatic detection of any peristomal skin involvement. The automatic detection of peristomal skin changes using a photograph enables remote care and speeds up treatment.

**Clinical relevance:**

The model developed using convolutional artificial neural networks is robust and reliable for detecting alterations in the peristomal skin, representing a significant advance in peristomal skin care for all ostomates, with early detection, prevention of complications and cost savings in treatment.

**Summary**
• Reporting method
◦ Checklist for Artificial Intelligence in Medical Imaging (CLAIM) was employed.
• Patient or public contribution
◦ By signing an informed consent form, patients agreed to have photographs taken of their peristomal skin and to be assessed by their stoma nurse.

## 1. Introduction

The creation of a stoma—a surgical opening connecting a hollow organ to the exterior of the body—is a common procedure for treating various diseases of the gastrointestinal and genitourinary tracts [1]. This intervention, whether temporary or permanent, represents a significant change in patients’ lives, impacting their daily routine and overall quality of life [2]. While ostomies are vital for managing conditions such as cancer, inflammatory bowel diseases, and others, long‐term stoma care can present considerable challenges, with peristomal skin complications (PSCs) being among the most frequent and significant problems [3, 4].

PSCs are the most common cutaneous problems observed following ostomy surgery, with an incidence ranging between 30% and 67% [1, 5]. Other studies have reported even higher incidence rates, reaching up to 73.4% [3]. The high prevalence of these complications not only negatively affects patients’ quality of life, causing pain, itching, bleeding, and discomfort, but also has a considerable economic impact on healthcare systems. PSCs have been shown to increase the length of hospital stay and readmission rates within 120 days postsurgery [3, 6]. Furthermore, it is estimated that the average person with an ostomy will experience some type of peristomal complication within 2 years of surgery [1].

The etiology of PSCs is multifactorial and complex. Irritant contact dermatitis is the most common complication and is caused by the exposure of the peristomal skin to stoma effluent. However, other causes can include infections (bacterial or fungal), exacerbated pre‐existing skin conditions, issues with the ostomy appliance, or the surgical technique itself [2, 4, 5]. It is crucial to recognize that healthcare professionals’ knowledge regarding these complications is often insufficient, underscoring the need for continuous training and a solid knowledge base, particularly for nurses who are typically the first point of contact in the care of these patients [2, 3].

Advanced practice nurses or those specialized in wound and ostomy care are essential resources for patients and healthcare systems. Their expertise is fundamental for the prevention and management of PSCs through patient education, proper appliance selection, and the implementation of simple yet effective interventions [6]. Despite the importance of these professionals and the growing need for specialized care, the availability of such resources is limited. This makes it even more imperative for all healthcare professionals to possess a basic understanding of PSCs to serve as the initial point of intervention for these problems [2].

Adequate knowledge and management of these complications are critical for improving the quality of life of ostomy patients and fostering a value‐based healthcare approach where patient education and empowerment are priority measures [3].

Artificial intelligence (AI) has the potential to enhance knowledge and optimize the management of PSCs. Specifically, AI resources that enable the analysis of peristomal skin images can offer nurses and other healthcare professionals assisted diagnosis and precise, immediate treatment recommendations, thereby facilitating faster and more standardized PSC management. A recent publication analyzing AI for stoma nurses concluded that studies on AI in ostomy care are scarce and found no applications specifically for peristomal skin [7].

Among AI techniques, those based on deep learning (DL) [8] yield the best results for image analysis. In the healthcare sector, a large volume of images is generated, leading to a notable increase in the use of DL on these images in recent years, achieving very positive outcomes. Among the DL models used, convolutional neural networks (CNNs) are prominent [9, 10]. Recent advances in medical image classification are based on CNNs and in comparative machine learning (ML) evaluations that optimize clinical imaging workflows [11]. Comparative studies of ML algorithms have proven highly effective in automated diagnosis by combining clinical and imaging data [12]. In cases such as brain tumor and skin cancer detection, DL models have outperformed traditional methods, providing high accuracy and real‐time processing capabilities for clinical workflows [13, 14]. Currently, it is being integrated as CNNs with Vision Transformers (ViT) to capture both fine‐grained local textures and global contextual relationships; these hybrid strategies have achieved good accuracy in colon and lung cancer detection [15, 16], while ViT‐based models have demonstrated great robustness in managing data imbalance through advanced augmentation techniques [17]. Although the most recent developments are these hybrid and comparative ML approaches with complex medical images, the application of AI in peristomal skin assessment has been very little developed. Regarding image studies associated with PSCs, to our knowledge, there is only one article [18] that applies DL‐based algorithms to detect changes in skin coloration.

The main objective of this study is to develop and validate a preliminary model based on CNNs for the binary classification of peristomal skin, enabling the distinction between healthy tissue and the presence of skin lesions, thereby laying the foundations for automated diagnostic systems. To achieve this, we will use proven, state‐of‐the‐art techniques and advanced CNNs that have been trained for this purpose.

## 2. Methods

### 2.1. Design

This study used a prospective design and is part of the research project Advances in the evaluation of peristomal skin in people with digestive elimination ostomies: Use of Artificial Intelligence Models by the Operational Programme European Regional Development Fund of Andalusia 2021–2027.

The target population for this research is people with elimination ostomies. It includes people with colostomy, ileostomy, or urostomy, across different ages, pathologies causing the ostomy (oncological and nononcological), and ostomy types within the three options described above.

The goal was to design a prospective study based on the use of state‐of‐the‐art CNNs, specifically ResNet50 [19], as a predictive model to identify peristomal skin alterations.

The Checklist for Artificial Intelligence in Medical Imaging (CLAIM) was used in the development of this study [20].

### 2.2. Data Collection

The data and images were collected by 24 stoma nurses from 17 hospitals participating in the study, who are part of the team. In the case of hospitalized patients, data were collected on the hospital ward. For patients attending for consultation, data were collected at scheduled checkup appointments. Sociodemographic and clinical data were collected from ostomates, and images of the peristomal skin were taken. For image acquisition, at least three photos of the stoma and peristomal skin were taken: one in a normal plane, one in a cropped plane, and one in a zenithal plane. A high‐resolution digital camera integrated into the latest‐generation mobile device was used, meeting the following technical requirements: 16 megapixels, f/2.0, dual‐tone flash, phase detection AF, auto HDR, RAW, and 4K video recording. To reduce variability in image capture, the stoma nurses were trained by the research team, and a data collection protocol was developed. In addition, a video tutorial on image acquisition was recorded in one of the university’s clinical simulation rooms with ostomy simulators in which the data collection procedure was reproduced.

In addition to the images, relevant sociodemographic and clinical data were collected by reviewing the patient’s medical history. A previous study recognized the need to complement the imaging assessment with this type of data [21]. A data collection notebook was created in electronic format on the SurveyMonkey platform. In case of data of interest not collected in the history, these were obtained by direct interview with the patient. To follow a homogeneous criterion that is easy for the work team to implement, the assessment register of the “Guide for planning and recording the care of the patient with an ostomy,” used by a large majority of hospital units, was adopted. The register includes the minimum criteria to be fulfilled by the patient with an ostomy based on functional health patterns (C O F, 2015). The data collection process took place from January to May 2025.

### 2.3. Image Tagging

The Discoloration, Erosion, Tissue overgrowth (DET) scale [22] was used to label the peristomal skin images. This is a standardized observational scale commonly used to assess EPP. It assesses the severity and extent of skin changes in three domains: discoloration, skin integrity, and tissue overgrowth. Each domain is assessed separately, with points awarded for the affected area (maximum 3 points) and the severity of involvement (maximum 2 points). The maximum score is 15 points. A score of 0 indicates intact skin; scores of 0–3 indicates mild skin impairment; 4‐7 indicates a moderate level of impairment; and 8–15 correspond to a high level of impairment. In this first phase of the study, images were binary categorized between intact skin (Score 0) and altered skin (Scores 1–15). This function was performed in pairs by three members of the research team with specialized training in ostomy care. In case of discrepancies, the third nurse intervened to reach a consensus. Specifically, in 35 cases, there were discrepancies in labeling, involving the third nurse in each case (between nurses 1 and 2: 12 cases, between nurses 2 and 3: 14 cases, and between nurses 1 and 3: 9 cases).

The minimum number of images needed to train a DL model depends on the model type, the required accuracy, and the problem being addressed. A previous study [23] analyzed the number of errors in a classifier as a function of the number of images used in training. The study was carried out with convolutional networks applied to classifying computed axial tomography images. To achieve an accuracy of 98%, they conclude that about 1000 images should be used for each class. For preliminary models such as the one developed in this study, fewer can be used. Based on the network of health centers collaborating with this project, the estimated number of cases to be assessed and the image acquisition process (minimum 3 per patient), 1000 images were deemed feasible, which is sufficient for the preliminary models to assess EPP states. This number exceeds previous studies on the application of AI models on EPP [18].

### 2.4. Ethical Considerations

For the conduct of this research, the standards of good clinical practice and the ethical principles established for research involving human beings were respected, in accordance with Law 14/2007, of July 3, on Biomedical Research. All the records were carried out in compliance with the precepts established in the current legislation on the protection of personal data contained in Organic Law 3/2018, of 5 December, on the Protection of Personal Data and the guarantee of digital rights, as well as in Law 41/2002, of 14 November, the basic law regulating patient autonomy and rights and obligations in the field of clinical information and documentation. The study has the approval of the Andalusian Biomedical Research Ethics Coordinating Committee (SICEIA‐2024–001022). All of the requirements have been followed in terms of informed consent forms, authorizations from the participating centers, and responsible declarations from the researchers forming part of the research team.

### 2.5. Statistical Analysis

#### 2.5.1. Exploratory Analysis

**FIGURE 1:**
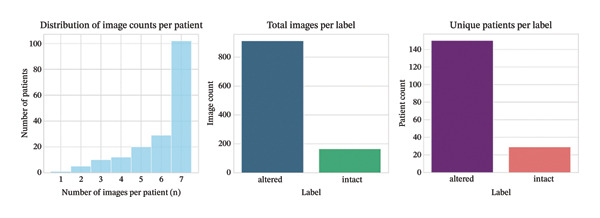
Imbalance affects the number of samples taken by patient (left), the global number of images per label (center), and the distribution of labels by patient (right).

In order to design a ML model capable of predicting from an image whether the peristomal skin has sustained damage, it is imperative to prepare the data from which the model will learn. The process begins with an exploratory analysis of the available data, from which the following key technical traits are obtained:

• File formats: The majority of the images were in the JPEG format, with a smaller subset employing the less prevalent Multipicture Object (MPO) Bitmap file format.
• Dimensions and orientation: The homogeneity pursued by the standardized protocol was not achieved. There was heterogeneity in resolution due to the varying characteristics of the devices used to take the photos (see Table 1). This also influenced the aspect ratio of the images, which determines the orientation (vertical or horizontal) used to take them.
• Data imbalance: The labels provided by the experts divided the dataset in two subsets: one with altered peristomal skin and the other with intact peristomal skin. The former accounted for more than 80.86% of the images (942 out of 1165), while the latter represented only 19.14% (223 images). This indicates that the imbalance ratio (IR) was 4.22, which is significantly different from the ideal value of IR = 1, representing a perfectly balanced dataset. Additionally, the number of pictures taken per patient shows a skewed distribution. While up to seven images were taken from a hundred patients, only four or fewer were obtained from 28 people. This hybrid imbalance, which affects the frequencies of images per class and patient, complicates data partitioning, as will be explained later. Figure 1 summarizes the imbalance statistics.

**TABLE 1:** Most common image dimensions and aspect ratio distribution.

Dimension	# Images	Aspect ratio	Percentage (%)
4032 × 3024	201	1.33	17.25
4000 × 3000	173	1.33	14.85
4080 × 3060	115	1.33	9.87
3000 × 4000	103	0.75	8.84
1200 × 1600	83	0.75	7.12
3072 × 4096	58	0.75	4.98
4624 × 3468	56	1.33	4.81
3472 × 4624	54	0.75	4.64
3024 × 4032	46	0.75	3.95
3264 × 2448	43	1.33	3.69
4096 × 3072	39	1.33	3.35
1600 × 1200	27	1.33	2.32
4624 × 3472	18	1.33	1.55
1536 × 2048	17	0.75	1.46
4160 × 3120	12	1.33	1.03

*Note:* The resolution of a digital image is measured in pixels and expressed as a product of the width and height dimensions. The aspect ratio is calculated as the ratio of width to height.

An exploratory analysis of the color distribution for each class of image was also conducted. While the images corresponding to unaltered skin exhibited a marginally more balanced distribution, characterized by a reduced intensity of red, the histograms appeared to lack sufficient information to differentiate between the two classes of images.

#### 2.5.2. Data Preparation

The preprocessing of image data constitutes a foundational step in ML workflows, with the objective of converting raw data into a format that is more amenable to analysis and model training. This process is imperative for the enhancement of model quality and efficiency.

##### 2.5.2.1. Image Rescaling and Normalization

The size of the input, defined as the total number of pixels in an image, directly affects the resources required to train a ML model. The number of parameters that must be adjusted increases in a linear, quadratic, or exponential manner, depending on the model’s architecture, in relation to the input size. Furthermore, it is essential that all samples provided to the model share the same dimensionality. Therefore, it is necessary to rescale the original images to standardize their size and reduce the number of attributes to those required by the selected model. The final size used in our case was 232 × 232, as imposed by the ResNet50 architecture.

Each pixel in the image is defined by three parameters following the RGB format, with integer values ranging from 0 to 255. The amplitude of each value is not the optimal input for a ML model, as most models require continuous values within the [0,1] interval. Therefore, a Z‐score normalization was applied to the data.

##### 2.5.2.2. Data Augmentation

To address the imbalance in our original dataset, where “altered” images significantly outnumber “intact” images, we applied various data augmentation techniques. Our goal was to achieve a more balanced distribution of images across the two classes to prevent the ML model from being biased toward the majority class. Standard oversampling methods, such as SMOTE, are typically applied to tabular data but rarely to images without first learning a representative embedding. This approach generally requires a substantial number of images from which the embedding can be learned. Likewise, implementing a synthetic image generation model, such as a generative adversarial network (GAN), demands a similar approach.

Therefore, we employed the most common techniques for image data augmentation, as used in studies such as Aslan and Özüpak [14]. It applies rotation, cropping, brightness adjustments, and other functions to magnetic resonance imaging (MRI) images. A similar approach was followed in Aslan and Özüpak [12] and Aslan and Özüpak [13]. As stated in Ozdemir et al. [16], the process of data augmentation has been shown to reduce the risk of overfitting of the trained models and bolster their robustness.

During the training phase, these techniques were applied exclusively to the minority class (“intact peristomal skin” images), while the test sets retained their original characteristics to ensure an unbiased evaluation.

According to the aforementioned restrictions, three additional samples were produced for each existing image by applying the following:

• Random rotation: A random rotation between 0 and 360° was applied to the images around their central point.
• Cropping and scaling: The peripheral area of the images was reduced by 25%, and the central region was scaled to maintain the original size.
• Contrast, brightness, and saturation: These techniques were applied to improve the quality of the augmented images by increasing the contrast by 30%, the brightness by 15%, and the saturation by 35%.

##### 2.5.2.3. Image Segmentation

**FIGURE 2:**
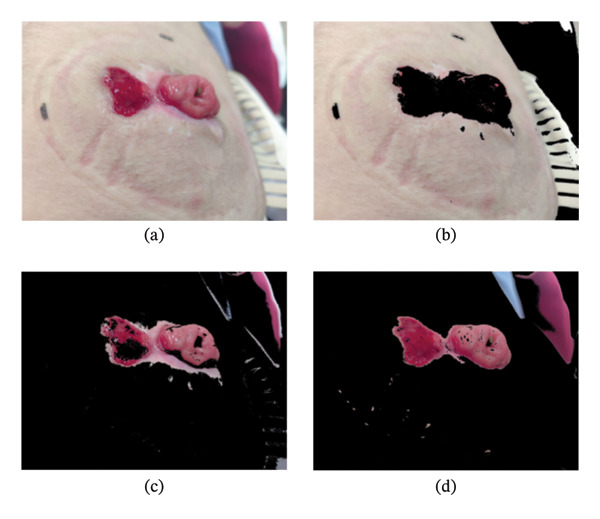
(a) Original image, (b) area around ostomy, (c) skin immediately surrounding the ostomy, and (d) only the ostomy.

Segmentation techniques allow us to highlight specific parts of an image depending on the applied filter. We defined three filters aimed at isolating the general area around the ostomy, the skin immediately surrounding it, and the ostomy itself (see Figure 2). These filters are applied only to a portion of the images to analyze how this technique can help the learning model.

We conducted other preliminary tests, such as using grayscaled images instead of color images, but we discarded them because they performed poorly.

##### 2.5.2.4. Data Partitioning Approaches

The partitioning approach, which involves dividing data into training, validation, and test subsets, plays a crucial role in evaluating the performance of predictive models. Hold‐out partitioning, where a fixed percentage of samples in the dataset is used for training and testing, is quite usual Aslan and Özüpak [12].

Cross‐validation is the most common approach when the sample size is small as is the case in this study. In some cases, fixed split and cross‐validation are combined [15]. The two usual options are 10‐fcv (folds‐cross‐validation) and 5‐fcv.

In cases where imbalance is detected, the implementation of stratified partitioning is strongly recommended. This approach ensures that the patterns within each subset align closely with the class distribution of the full dataset. Therefore, the imbalance level identified in the training split is consistent with that of the test split. Samples are typically selected at random by each class. The training subset can then undergo data augmentation to achieve class balance, while the test subset remains untouched. This approach, combined with 10‐fcv or 5‐fcv, is the one found in most published studies.

Although stratified partitioning at the class level is almost the standard, there are concerns that it can produce data leaks resulting in artificial higher performances of the trained models. According to Young et al. [24], this outcome would result from the utilization of images of the same patient, divided between test and training subsets. The most effective solution is to ensure that images from the same patient are not used across training and test subsets. However, this approach complicates the partitioning procedure, especially in cases when data imbalance is present and when the dataset is not sufficiently large. Consequently, the performance of models evaluated in this manner is consistently inferior. As stated in the review made by Young et al. [24], experiments that do not separate individuals’ data achieve an accuracy rate as high as 99% (mean 97.1%) and an area under the ROC curve (AUC) between 96% and 99%. Conversely, studies that implement this distinction yield lower outcomes, with accuracy as low as 66% (mean 78.5%) and AUC in the range 75%–93%.

Given the modest size of our dataset, its significant class imbalance, and the uneven distribution of samples across patients, it is clear that the most effective approach to assessing model performance would be through stratified partitioning with cross‐validated evaluation. As previously mentioned, this choice has the potential to lead to inflated results compared with a real case scenario. To this end, we have conducted our analysis with a dual experimental framework:

• FR1: First, the data were partitioned following a stratified 10‐fcv, the training split was data augmented, and a ResNet50 model was trained. An independent test set was used to assess performance.
• FR2: Second, the data were partitioned so that all samples from the same individual were either in the training or test subset. The procedure to assess performance was similar to the one described in FR1, but three different models were trained and evaluated in order to achieve the best possible outcome.

In the following, please note that differences between configurations and results will be noted where needed, depending on the experimental framework.

#### 2.5.3. Predictive Models

We approached the classification of peristomal skin images using state‐of‐the‐art, pretrained CNNs. This choice was motivated by their proven efficacy and superior efficiency compared with other existing methods. Initially, InceptionV3 [25] was selected as the base model. This architecture was chosen for its ability to capture data variability effectively without consuming significant resources. ResNet50 was also subsequently evaluated. ResNet50 [19] was considered because it has only two more layers than InceptionV3 and is effective in mitigating the vanishing gradient problem. This capability was deemed potentially beneficial for distinguishing subtle visual characteristics often present in highly specific medical images, such as those of peristomal skin. Finally, DenseNet121 [26] was also considered. In this model, each layer receives a concatenation of the outputs from the previous layers (dense connectivity). This trait mitigates the vanishing gradient problem, but it does so at the cost of higher memory needs.

To adapt these pretrained models to specific tasks, we applied transfer learning and fine‐tuning techniques. This process included loading the models with pretrained weights, freezing all base model parameters, and replacing the original output layer with a layer suitable for binary classification. Then, only the modified final layer was selectively unfrozen and trained. This strategy enabled the models to retain the low‐level features learned from large datasets while adapting to the unique characteristics of peristomal skin images.

All the steps in the study were implemented in Python. OpenCV and NumPy libraries were used to preprocess the images, PyTorch to run the models, and Scikit‐learn to compute the evaluation metrics.

#### 2.5.4. Validation

As mentioned, the problem addressed in this paper is a binary classification task. In this type of problem, one class is known as the positive class and the other as the negative class. In this context, true positive (TP) and true negative (TN) refer to the number of correctly classified examples of the positive and negative classes, respectively. Similarly, false positive (FP) and false negative (FN) refer to the number of examples misclassified for each class.

The performance of the classification models was assessed using three well‐known measures, the definition of which is based on the aforementioned concepts:

• Accuracy: this represents the proportion of examples that were correctly classified.
(1) Accuracy=TP+TN,TP+FN+FP+TN.
• F1‐score: this is a combination of two measures: the proportion of positive examples that are correctly classified, and the proportion of examples that are correctly classified as positive out of the total number that are classified as positive.
(2) F1–score=2∗TP2∗TP+FP+FN.
• AUC: the AUC is a metric that effectively measures the model’s ability to distinguish between negative and positive examples. The ROC curve plots the true‐positive rate (TPR) versus the false‐positive rate (FPR).

## 3. Results

**FIGURE 3:**
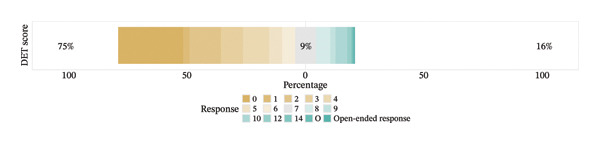
DET scale score in the study sample.

The 1165 images included in this research belong to 189 patients with ostomies, whose most relevant demographic and clinical characteristics are shown in Table 2. The sample of patients included had varied peristomal skin conditions measured by the DET scale, as shown in Figure 3. In 35 cases, there were discrepancies in labeling, involving the third nurse in each case (between nurses 1 and 2: 12 cases, between nurses 2 and 3: 14 cases, and between nurses 1 and 3: 9 cases).

**TABLE 2:** Demographic and clinical characteristics of the patients in the sample.

		***N* of subjects**	**%**	**Median (IQR)**

Gender	Female	76	40.2	
	Male	113	59.3	

Age (years)				67.0 (16.0)
	Female			67.0 (17.0)
	Male			67.0 (15.0)

Ostomy	Colostomy	102	53.68	
	Female	51	67.10	
	Male	51	45.53	
	Ileostomy	72	37.89	
	Female	23	30.26	
	Male	49	43.75	
	Urostomy	14	7.37	

	Female	2	2.63	
	Male	12	10.71	
DET scale score				3.0 (6.0)
	Intact skin	50	26.47	
	Damaged skin	139	73.54	

*Note:* DET: Discoloration, Erosion, Tissue overgrowth; IQR: interquartile range.

This section describes the results obtained from experiments, including accuracy, F1‐score, and AUC for each tested configuration. Subsection 3.1 corresponds to the FR1 experimental framework, while Subsection 3.2 shows the outcomes from FR2.

### 3.1. First Experimental Framework

A ResNet50 model was trained using 30 epochs, a batch size of 32, and an early stopping technique. A stratified 10‐fcv methodology was applied.

Table 3 shows the results of the proposed models in three different scenarios. These scenarios consider whether the segmentation technique has been applied to training images and, if so, whether it has been applied to 20% or 80% of the images. The model configuration parameters for the three scenarios are discussed in the previous sections.

**TABLE 3:** Performance metrics for each tested configuration.

Segmentation	Accuracy	F1‐score	AUC
0%	0.878	0.875	0.908
20%	0.889	0.890	0.924
80%	0.867	0.867	0.910

As can be seen in Table 3, the lowest value obtained is 0.867 for accuracy and F1‐score metrics using 80% segmented images in model training. However, for the AUC measure, the lowest value is 0.908, obtained by the model trained without image segmentation. Nonetheless, for the three experimentation scenarios tested, the models obtained good results for all metrics considered. The model trained using 20% of the segmented images reached the best results for the three metrics: 0.889 for accuracy, 0.890 for the F1‐score, and 0.924 for AUC.

The three models achieved high and similar accuracy and F1‐score values, demonstrating that they perform consistently well in predicting both classes and are free of bias. The high values achieved in F1‐score indicate that the models correctly classify a high proportion of images of altered skin and minimize two types of errors: classifying images of intact skin as altered skin (FN) or classifying images of altered skin as intact skin (FP).

**FIGURE 4:**
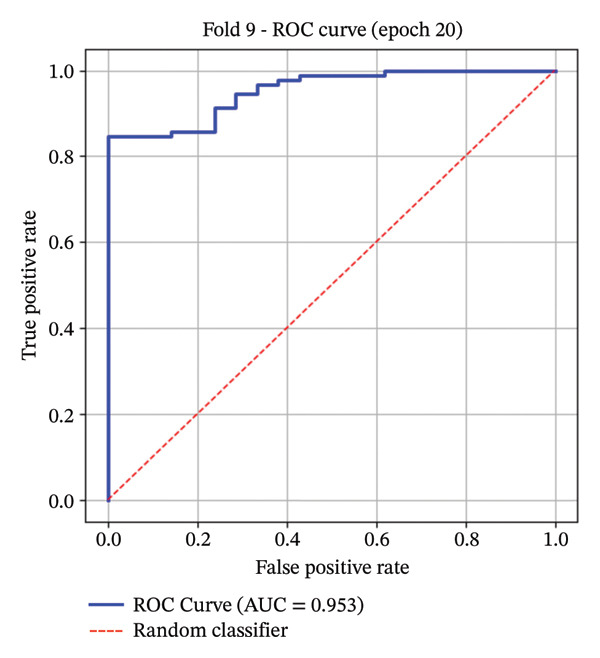
AUC curve for ResNet50 using 20% segmentation.

The good performance of the models can also be deduced by considering the AUC measure, for which values above 90% are obtained in all scenarios. The ROC curve represented in Figure 4 shows that the model achieves a high TPR, indicating that it correctly classifies images of skin alterations. Meanwhile, it maintains a low FPR, indicating a low rate misclassifying intact skin as altered skin.

In summary, the models perform robustly and reliably in both skin‐altered and skin‐intact image classification. This can be deduced regarding that accuracy and F1‐score reach high and similar values and also the AUC metric is even better.

To statistically validate the results, a Student′s *t*‐test, with a 95% confidence level, was applied to the best‐performing scenario, in which 20% of the segmented images were used for training purposes. The confidence interval for the F1‐score measure ranged from 0.8696 to 0.91 (95% CI = 0.8596, 0.9100) and from 0.9025 to 0.9452 for the AUC metric (95% CI = 0.9025, 0.9452). This shows that the values are close to 90% for F1‐score and nearly 95% for AUC. Therefore, it is clear that the model is highly reliable.

Table 4 shows the mean and standard deviation of the number of errors made by the model, as well as the breakdown of these errors into FP and FN.

**TABLE 4:** Statistical measures of errors.

	**Errors (%)**	**FN (%)**	**FP (%)**

Mean	13.1	8.8	30.3
Standard deviation	2.8	2.1	7.9

### 3.2. Second Experimental Framework

In this case, the data were partitioned ensuring that images from the same patient were fully assigned either to the test or the train split. Three different models were trained, increasing the number of epochs to 100 while maintaining the batch size. The loss function was also adjusted, using BinaryMCC instead of cross entropy.

Average values and standard deviations for each model and metric are shown in Table 5. As can be seen, even for InceptionV3—which is the best one with this framework—accuracy and AUC are clearly worse to the values resulted from the common stratified partitioning, while F1‐score is quite similar.

**TABLE 5:** Results for each CNN model and metric from data partitioned ensuring that images from the same individual go only to train or test split.

Model	Accuracy	F1‐score	AUC
ResNet50	0.737 ± 0.046	0.838 ± 0.033	0.630 ± 0.050
DenseNet121	0.744 ± 0.052	0.842 ± 0.036	0.643 ± 0.112
InceptionV3	0.778 ± 0.058	0.868 ± 0.038	0.653 ± 0.120

**FIGURE 5:**
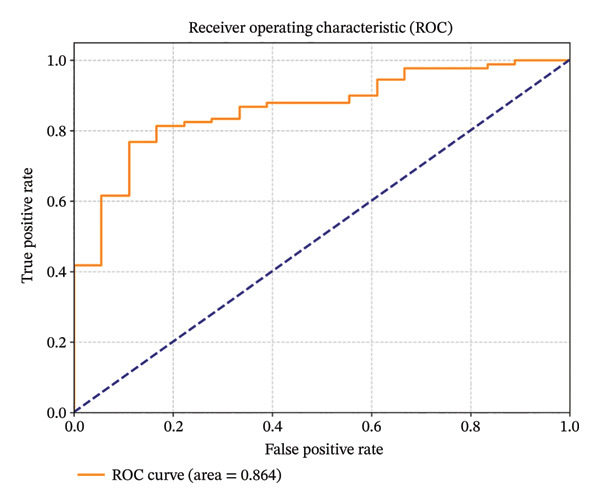
AUC curve for InceptionV3.

Forcing the separation of individuals between splits produces a high variability in results, mainly in the AUC metric. Although mean AUC is 0.653, it goes slightly below in some folds and also notably above in others, as can be seen in Figure 5.

As previously, the confidence intervals for the best model were obtained with a 95% level. The one for accuracy ranged from 0.7363 to 0.8198, for F1‐score was in the interval from 0.8404 to 0.8948, and for AUC from 0.5669 to 0.7387. The higher deviation for AUC has a clear impact in the confidence values for this metric.

## 4. Discussion

The profile of the sample found is similar to that described in the most recent literature, with males in just over half of the cases and aged 60–70 years [21, 27]. More than half of ostomies are colostomies, followed by just over a third ileostomies, and the rest are urostomies [27, 28]. Our study also found a similar proportion of patients with peristomal skin damage, which falls within 65%–75% of the cases [21, 27–29]. On the other hand, the robust and reliable model for detecting altered peristomal skin, with an accuracy (calculated by TPs and TNs on the total sample) greater than 0.8 in all cases, which is higher than that found by [21] (0.61) and similar to another study carried out with AI on ostomies, even if the objectives were very different [30], where the area under the curve is the same (0.9). The use of peristomal skin images to assess their condition, diagnose peristomal skin alterations, and establish specific treatment has been used as a new opportunity in the care of these patients, by providing information that goes beyond the written description of the peristomal skin and allows remote care [21, 31, 32]. In addition, with the use of peristomal imaging, a high level of inter‐ and intraobserver agreement of expert nurses has been found for remote assessment, especially for the identification of clinical signs (incrustations, nodules, and mucocutaneous separation) and diagnoses (such as allergic contact dermatitis) [21, 32].

To further overcome the subjective assessment of the visual inspection of peristomal skin by stoma nurses, manage time, and avoid regular face‐to‐face consultations, assessing peristomal skin with AI can be a breakthrough. With our research, the models generated with CNNs from 1165 images of peristomal skin allowed us to predict, with an approximate accuracy of 90%, peristomal skin with some type of alteration. These results are very similar to those obtained by [18] who demonstrated the feasibility of implementing AI algorithms for an objective and consistent assessment of changes in the peristomal skin, specifically, by training two models based on CNNs. The first the discoloration model, from 614 images, achieved 95% accuracy in predicting the area of reddened skin and the second, the leak model, determined the leak area with 98.8% accuracy using 954 images of products used to detect leak patterns. Even more recently, Reference [33], using advanced ConvNeXt‐type CNN models with 825 images, obtained relatively strong accuracy for the identification of irritant dermatitis (around 0.7) and intact skin although the models showed limitations in recognizing other complications of the peristomal skin. Recently, other studies using CNNs to diagnose health conditions from medical images have also achieved an accuracy rate of over 90%, both for skin conditions, as skin cancer (accuracy of 92.97%) [13] and other types of cancer, as colon cancer (accuracy of 96,2%) [15] and lung cancer (accuracy of 99.54%) [16]. It is justified our methodological choice to employ robust, pure CNNs (such as ResNet50) rather than the hybrid CNN–Transformer strategies [15, 17] because although hybrid models and complex comparative ML workflows are optimal for some diagnostic fields [12, 14], peristomal skin assessment is still in its early stages. This enables the establishment of a highly reliable, computationally efficient, and fundamental system, designed specifically for ostomy nursing care.

**FIGURE 6:**
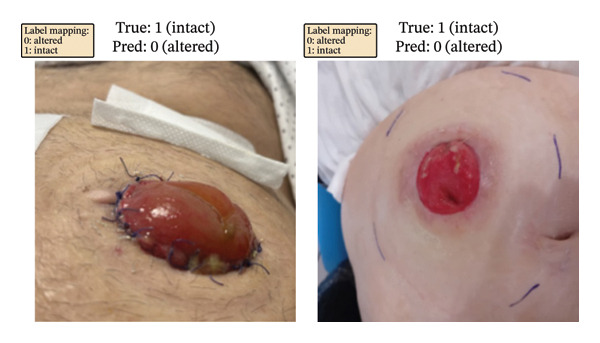
False positives.

The quality of the images used to train an AI model directly impacts its subsequent behavior when classifying new samples. These images must contain only the peristomal skin, with shadows avoided, and the stoma centered. Our proposed model produces a certain number of FPs. We have thoroughly analyzed these cases to draw sound conclusions. Figure 6 shows two representative examples. The image on the left shows a stoma following a recent operation, with stitches still visible around it. The image clearly shows gauze dressing at the top and on the left. These elements confuse the model and cause it to misinterpret the skin, indicating a lesion. The problem with the image on the right is more subtle. The skin surrounding the stoma is slightly reddened, much more so than the rest of the peristomal skin. Even experts recognize cases like this as being on the borderline between completely healthy skin and skin with a mild condition. The model suggests a potential problem here.

**FIGURE 7:**
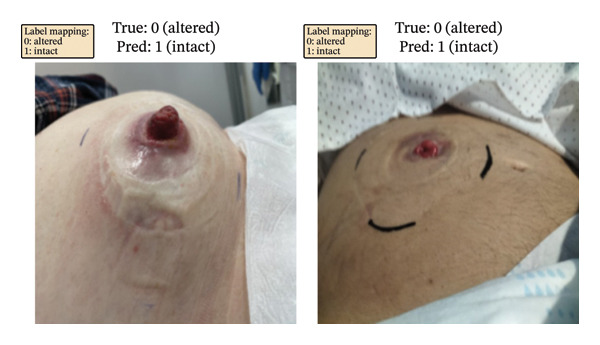
False negatives.

While the number of FNs is significantly lower in our model, we have thoroughly analyzed these cases. Figure 7 shows two examples of this kind of misclassification. It is clear that both have some common traits, such as the presence of other elements aside from the peristomal image (bedding, what appears to be a door handle, clothing in the corner of the left image, etc.), as well as shadows covering parts of the images. Another issue to consider is that the stoma is not centered, and the photo angle is not entirely overhead. This latter aspect could hinder the model’s ability to adequately view parts of the peristomal skin, while the human evaluator was able to see these areas from different angles. Furthermore, there is a significant imbalance in the number of images in the two classes of interest. Other issues arise during model training, such as the presence of irrelevant elements and uneven sample distribution across patients.

To determine the reliability of the obtained models, one can apply any of the explainable artificial intelligence (XAI) techniques. Here, we will use Grad‐CAM [34], one of the most well‐known techniques for generating visual explanations. This technique produces a heatmap indicating the areas of the image on which the model focuses to make its prediction. The importance of the areas in the heatmap is marked from red to blue in descending order.

**FIGURE 8:**
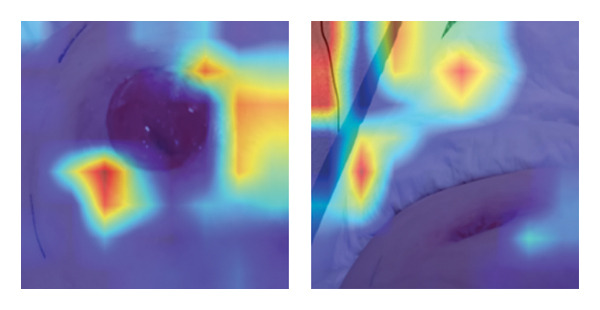
Result of applying Grad‐CAM.

Two images have been selected—one correctly classified and the other misclassified—by the InceptionV3 model, which has achieved the best prediction results. Figure 8 shows an image of intact skin on the left that was correctly classified by the model. As can be seen in the heat map, the areas on which the model focuses correspond to the peristomal skin. In contrast, the image on the right shows a patient with altered skin that was misclassified by the model. The explainability method reveals that, when making its prediction, the model is focusing not on the peristomal skin but on external elements that do not belong to the patient’s skin. This supports the previously mentioned point about the importance of a high‐quality training dataset containing only the elements essential for proper model training, ensuring that models focus solely on the elements important for prediction—in this case, the peristomal skin area.

It is imperative to note that training large vision models typically requires collecting a substantial number of images, often ranging from hundreds of thousands to millions, to ensure comprehensive training. The team expended considerable effort compiling a sufficient number of images; however, this number falls considerably short of the required quantities. The applied methodology, based on a pretrained model fine‐tuned on these images, along with the preprocessing steps used to improve the learning process, has enabled us to obtain remarkably good results.

A significant challenge was the aforementioned imbalance between the two considered classes, with a paucity of images of unaltered skin. The conducted data augmentation, which produces additional samples by applying affine transformations, has proven to be highly effective.

The DET scale used in the present study for image labeling is a widely validated clinical practice used in key studies [35], as well as the SACS scale [36]. The high prevalence of altered skin (73.5%) in the present study is consistent with that reported by other authors: 73% [36]. However, the subjectivity shown by the DET scale [32] has been recently confirmed by [37] due to the low‐quality evidence regarding the reliability and content validity (of both the DET and the SACS). The variability and subjectivity inherent in human evaluation through scales contrasts with the high precision achieved with our model, even though it has been trained with data that possess this human variability.

However, the assessment of stomas is moving toward hybrid models that combine visual evaluation with patient‐reported outcomes [38] so that any diagnosis based solely on photos is limited and requires clinical context [21]. Therefore, models should evolve to integrate patient symptoms, aligning with the advanced practice proposed by [38, 39].

## 5. Conclusions

By processing peristomal skin images with AI, we have developed robust, reliable preliminary models for detecting altered peristomal skin, enabling us to predict the extent of peristomal alteration with high confidence. However, subsequent advancements in the AI model development could encompass several domains. First, the classifier could be extended to consider a finer granularity in classifying images, not being limited to separating affected and unaffected skin only. The imbalance problem can also be addressed by leveraging synthetic sample creation through generative models, but a larger number of images would be needed. Implementing these transformations would introduce greater variability than that achievable with affine transformations alone. Finally, it would be worthwhile to assess the viability of compressing the AI model for deployment on mobile devices.

Regarding future lines of research, it should be considered that further progress should be made in analyzing the data set that influences the condition of the peristomal skin, in line with what has been discussed above. The combination of image features with structured sociodemographic and clinical data will contribute to improving the sensitivity and specificity of the AI model for the classification and prediction of the severity of peristomal skin involvement. Along these lines, more precise analyses of the state of this skin are also necessary, taking advantage of the analysis domains offered by scales such as the DET scale, an aspect on which this research team continues to work. Another future line of interest concerns validating the AI tool, which requires progress in assessing its reliability and agreement with diagnoses issued by specialized clinical personnel. For example, carrying out interobserver concordance studies to measure the agreement between the diagnosis of AI and that of the independent clinical judgment of experts (nurses and dermatology specialists, among others), both in the identification of the presence or absence of alterations in the peristomal skin and in the classification of their severity. Another advance in this line concerns the development of more research on image processing that enables the identification of specific lesions that may appear on this type of skin. In addition, the algorithm could be optimized to move from a diagnostic tool (as proposed in this study) to a predictive tool and even to one that could offer a treatment response based on the condition of the skin. Finally, another future line concerns the implementation of this type of AI model in mobile applications or other digital systems that enable telemonitoring of the peristomal skin in ostomy patients.

From our study, we can bring great potential in clinical practice, as it allows patients and stoma nurses to monitor the condition of the peristomal skin by automatically detecting even small changes in discoloration. In this way, early detection and prevention of further complications are facilitated, positively impacting the patient’s health‐related quality of life. Likewise, automatic detection of peristomal skin alterations in photos avoids face‐to‐face appointments and facilitates timely care, which is essential for rapid intervention in PSCs.

Subsequent advancements in the AI model development could encompass several domains. First, the classifier could be extended to consider a finer granularity in classifying images, not being limited to separating affected and unaffected skin only. The imbalance problem can also be addressed by leveraging synthetic sample creation through generative models. Implementing these transformations would introduce greater variability than that achievable with affine transformations alone. Finally, it would be worthwhile to assess the viability of compressing the AI model for deployment on mobile devices.

The proposed model shows great promise for integration into computer‐assisted care for ostomy patients. It is worth highlighting its potential as a tool for real‐time decision‐making support, as it has the potential to reduce interobserver variability and the workload of nurses.

This study represents an important step toward the accurate and efficient diagnosis of PSCs, contributing to the advancement of healthcare solutions based on AI.

## Author Contributions

Isabel María López‐Medina: conceptualization, methodology, writing–original draft, writing–review and editing, and project administration; César Hueso‐Montoro: conceptualization, methodology, writing–original draft, and writing–review and editing; Francisco Charte‐Ojeda: conceptualization, methodology, validation, formal analysis, writing–original draft, and writing–review and editing; Carmen Álvarez‐Nieto: methodology, data curation, writing–original draft, and writing–review and editing; José Pablo Soriano‐Torres: methodology, validation, and formal analysis; Francisco Pedro García‐Fernández: investigation, writing–original draft, and writing–review and editing; Concepción Capilla‐Díaz: investigation and writing–review and editing; Ana Carmen Montesinos‐Gálvez: investigation and writing–review and editing; Noelia Moya‐Muñoz: investigation and writing–review and editing; Claudia Cuevas‐Sánchez: investigation and writing–review and editing; María Dolores Pérez‐Godoy: conceptualization, methodology, validation, formal analysis, writing–original draft, writing–review and editing, and project administration.

## Funding

This study was funded by the Operational Programme European Regional Development Fund of Andalusia 2021–2027, M.1.B.BTA_000115_UJA23, MICIU/AEI/10.13039/501100011033, and by the ERDF, EU, PID2023‐149511OB‐I00.

## Disclosure

All authors have reviewed and approved the final version of the manuscript.

## Conflicts of Interest

The authors declare no conflicts of interest.

## Supporting Information

Additional supporting information can be found online in the Supporting Information section.

## Supporting information


**Supporting Information** Checklist for Artificial Intelligence in Medical Imaging (CLAIM) used in the development of this study.

## Data Availability

The data that support the findings of this study are available on request from the corresponding author. The data are not publicly available due to privacy or ethical restrictions.
