# Supplementary material for: Identification of Peristomal Skin Alterations Using Convolutional Artificial Neural Networks
Source: Nurs Res Pract. 2026 Jun 14;2026:2927548. doi: 10.1155/nrp/2927548 (PMC13266281; doi:10.1155/nrp/2927548)
Supplement: Supplementary file 1 — Supporting Information Checklist for Artificial Intelligence in Medical Imaging (CLAIM) used in the development of this study. [file NRP-2026-2927548-s001.docx]

**Checklist for Artificial Intelligence in Medical Imaging (CLAIM): 2024 Update**

| Section / Topic | No. | Item | Page / Line | No | NA |
| --- | --- | --- | --- | --- | --- |
| TITLE / ABSTRACT |  |  |  |  |  |
|  | **1** | Identification as a study of AI methodology, specifying the category of technology used (e.g., deep learning) | 3 | 66 |  |
| ABSTRACT |  |  |  |  |  |
|  | **2** | Summary of study design, methods, results, and conclusions | 3 | 69-100 |  |
| INTRODUCTION |  |  |  |  |  |
|  | **3** | Scientific and/or clinical background, including the intended use and role of the AI approach | 4  5 | 144-148  149-172 |  |
|  | **4** | Study aims, objectives, and hypotheses | 5 | 173-177 |  |
| METHODS |  |  |  |  |  |
| *Study Design* | **5** | Prospective or retrospective study | 5 | 181 |  |
|  | **6** | Study goal | 5 | 188-190 |  |
| *Data* | **7** | Data sources | 6 | 194-216 |  |
|  | **8** | Inclusion and exclusion criteria |  |  | X |
|  | **9** | Data pre-processing | 9 | 294-358 |  |
|  | **10** | Selection of data subsets | 10 | 360-400 |  |
|  | **11** | De-identification methods |  |  | X |
|  | **12** | How missing data were handled |  |  | X |
|  | **13** | Image acquisition protocol | 6 | 198-206 |  |
| *Reference Standard* | **14** | Definition of method(s) used to obtain reference standard |  |  | X |
|  | **15** | Rationale for choosing the reference standard |  |  | X |
|  | **16** | Source of reference standard annotations |  |  | X |
|  | **17** | Annotation of test set |  |  | X |
|  | **18** | Measures of inter- and intra-rater variability of features described by the annotators |  |  | X |
| *Data Partitions* | **19** | How data were assigned to partitions | 11 | 391-397 |  |
|  | **20** | Level at which partitions are disjoint | 11 | 391-397 |  |
| *Testing Data* | **21** | Intended sample size | 4 | 150-160 |  |

| Section / Topic | No. | Item | Page / Line | No | NA |
| --- | --- | --- | --- | --- | --- |
| *Model* | **22** | Detailed description of model | 11 | 403-415 |  |
|  | **23** | Software libraries, frameworks, and packages | 12 | 422-424 |  |
|  | **24** | Initialization of model parameters | 13 and 15 | 464-465 and 515-517 |  |
| *Training* | **25** | Details of training approach | 11, 13  and 15 | 386-397  464-469  515-517 |  |
|  | **26** | Method of selecting the final model |  |  | X |
|  | **27** | Ensembling techniques |  |  | X |
| *Evaluation* | **28** | Metrics of model performance | 12 | 427-446 |  |
|  | **29** | Statistical measures of significance and uncertainty | 15 | 495-511 |  |
|  | **30** | Robustness or sensitivity analysis | 11, 15 and  15 | 386-397, 495-511 and  514-515 |  |
|  | **31** | Methods for explainability or interpretability | 18 | 612-616 |  |
|  | **32** | Evaluation on internal data | 11 | 391-397 |  |
|  | **33** | Testing on external data |  |  | X |
|  | **34** | Clinical trial registration |  |  | X |
| RESULTS |  |  |  |  |  |
| *Data* | **35** | Numbers of patients or examinations included and excluded | 12 | 449-456 |  |
|  | **36** | Demographic and clinical characteristics of cases in each partition | 12 | 450-458 |  |
| *Model performance* | **37** | Performance metrics and measures of statistical uncertainty | 15 | 495-511 |  |
|  | **38** | Estimates of diagnostic performance and their precision | 13-16 | 464-536 |  |
|  | **39** | Failure analysis of incorrect results | 17 | 583-608 |  |
| DISCUSSION |  |  |  |  |  |
|  | **40** | Study limitations | 17  18 | 584-593  598-608 |  |
|  | **41** | Implications for practice, including intended use and/or clinical role | 19 | 631-654 |  |
| OTHER INFORMATION |  |  |  |  |  |
|  | **42** | Provide a reference to the full study protocol or to additional technical details | 2 | 57 |  |
|  | **43** | Statement about the availability of software, trained model, and/or data | 2 | 38-40 |  |
|  | **44** | Sources of funding and other support; role of funders | 2 | 54-62 |  |

* Indicate page and/or line number for each checklist item that is present. NA = not applicable.
